# Combining Individual‐Based Radio‐Tracking With Whole‐Genome Sequencing Data Reveals Candidate for Genetic Basis of Partial Migration in a Songbird

**DOI:** 10.1002/ece3.70800

**Published:** 2025-01-09

**Authors:** Matthias H. Weissensteiner, Kira Delmore, Valentina Peona, Juan Sebastian Lugo Ramos, Gregoire Arnaud, Julio Blas, Bruno Faivre, Ivan Pokrovsky, Martin Wikelski, Jesko Partecke, Miriam Liedvogel

**Affiliations:** ^1^ Institute of Avian Research “Vogelwarte Helgoland” Wilhelmshaven Germany; ^2^ Max Planck Research Group Behavioural Genomics Max Planck Institute for Evolutionary Biology Plön Germany; ^3^ Department of Ecology Evolution and Environmental Biology Columbia University New York New York USA; ^4^ Vogelwarte Sempach Switzerland; ^5^ Department of Genetics and Bioinformatics Swedish Natural History Museum Stockholm Sweden; ^6^ Neural Circuits and Evolution Laboratory The Francis Crick Institute London UK; ^7^ Centre d'Ecologie Fonctionnelle et Evolutive (CEFE) Univ Montpellier, CNRS, EPHE, IRD Montpellier France; ^8^ Department of Conservation Biology and Global Change Estación Biológica de Doñana (EBD—CSIC) Sevilla Spain; ^9^ UMR CNRS BioGéoSciences Université de Bourgogne Dijon France; ^10^ Department of Migration Max Planck Institute of Animal Behavior Radolfzell Germany; ^11^ Department of Biology and Environmental Sciences Carl von Ossietzky Universität Oldenburg Oldenburg Germany

**Keywords:** genomics, partial migration, period gene, population genetics, songbird

## Abstract

Partial migration is a phenomenon where migratory and resident individuals of the same species co‐exist within a population, and has been linked to both intrinsic (e.g., genetic) as well as environmental factors. Here we investigated the genomic architecture of partial migration in the common blackbird, a songbird that comprises resident populations in the southern distribution range, partial migratory populations in central Europe, and exclusively migratory populations in northern and eastern Europe. We generated whole‐genome sequencing data for 60 individuals, each of which was phenotyped for migratory behavior using radio‐telemetry tracking. These individuals were sampled across the species' distribution range, including resident populations (Spain and France), obligate migrants (Russia), and a partial migratory population with equal numbers of migratory and resident individuals in Germany. We estimated genetic differentiation (F_ST_) of single‐nucleotide variants (SNVs) in 2.5 kb windows between all possible population and migratory phenotype combinations, and focused our characterization on birds from the partial migratory population in Germany. Despite overall low differentiation within the partial migratory German population, we identified several outlier regions with elevated differentiation on four distinct chromosomes. The region with the highest relative and absolute differentiation was located on chromosome 9, overlapping *PER2*, which has previously been shown to be involved in the control of the circadian rhythm across vertebrates. While this region showed high levels of differentiation, no fixed variant could be identified, supporting the notion that a complex phenotype such as migratory behavior is likely controlled by a large number of genetic loci.

## Graphical abstract

Combining individual‐based radio‐tracking and whole‐genome sequencing, we investigated the population genetics of a partial migratory songbird, the common blackbird. Using population genetic summary statistics, we identify a region associated with migratory behavior in the vicinity of the period 2 gene.

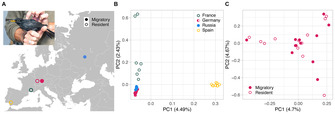

## 1 Introduction

Bird migration is one of the most fascinating and best‐studied behavioral phenomena in the animal kingdom. While patterns of variation in migratory behavior and its ecological and evolutionary causes have been investigated in great detail, its genetic and physiological control remains understudied, limiting our understanding of the proximate control of migration, and its evolutionary potential (Helbig 1996; Plummer et al. 2015; Pulido 2011). Migration can vary drastically between species, among populations of the same species (e.g., Berthold et al. 1992), and among individuals within populations (Hegemann, Fudickar, and Nilsson 2019; Fudickar et al. 2013). In numerous species, including the common blackbird, *Turdus merula* , the propensity to migrate as well as the distance traveled differs with latitude, and many species include partial migratory populations, that is, populations where only some individuals migrate, while others stay year‐round (Pulido 2011; Hegemann, Fudickar, and Nilsson 2019; Linek et al. 2021). Quantitative genetics analyses of selection and cross‐breeding experiments under controlled laboratory conditions clearly showed a heritable component of migratory traits (Berthold et al. 1992; Helbig 1991; Helbig 1996; Pulido and Berthold 2010), but the genomic architecture underlying this behavior remains poorly understood. In order to investigate the molecular basis of a complex trait such as migration, the behavior needs to be accurately measured and characterized. Migratory flights may cover thousands of kilometers across and beyond continents, making it difficult to accurately record the focal behavior or trait in a natural setting. Much of our current understanding of the molecular basis of bird migration is based on phenotype characterization using indirect measures, most notably captive breeding schemes and quantitative genetics analyses on few study species (e.g., the blackcap, (Berthold and Querner 1981)), displacement experiments (Perdeck 1958; Thorup et al. 2007; Chernetsov, Kishkinev, and Mouritsen 2008), ringing recovery analyses (Mettler et al. 2013), and studies using stable isotope signatures wintering location (Bensch et al. 2009; Lundberg et al. 2017). Importantly, these measures are approximations (proxies) for the behavior in focus, and are mostly based on population‐based averages, which especially in the case of partially migratory populations, where individuals within the same population exhibit different phenotypes, precludes the characterization of individual behavior. However, recent advances in tracking technology and weight reduction of tagging devices are increasingly permissive of the characterization of migration behavior at the individual level with increasing temporal and spatial resolution (Jetz et al. 2022). Much of this research has focused on characterizing variable orientation behavior across migratory divides, that is, areas where neighboring populations breed in close vicinity, but follow distinct migratory directions (e.g., Delmore and Irwin 2014; Delmore et al. 2016; Delmore et al. 2020; Sokolovskis et al. 2019). Studies on the molecular basis of other migratory traits, such as propensity to migrate especially in the context of partial migration (Hegemann, Fudickar, and Nilsson 2019; Hegemann, Marra, and Tieleman 2015), have so far been scarce; a transcriptomic study on a partial migratory population of the common blackbird detected four differentially expressed genes between migrants and residents which may play roles in determining the decision to migrate, or control physiological processes required for migration (Franchini et al. 2017).

**FIGURE 1:**
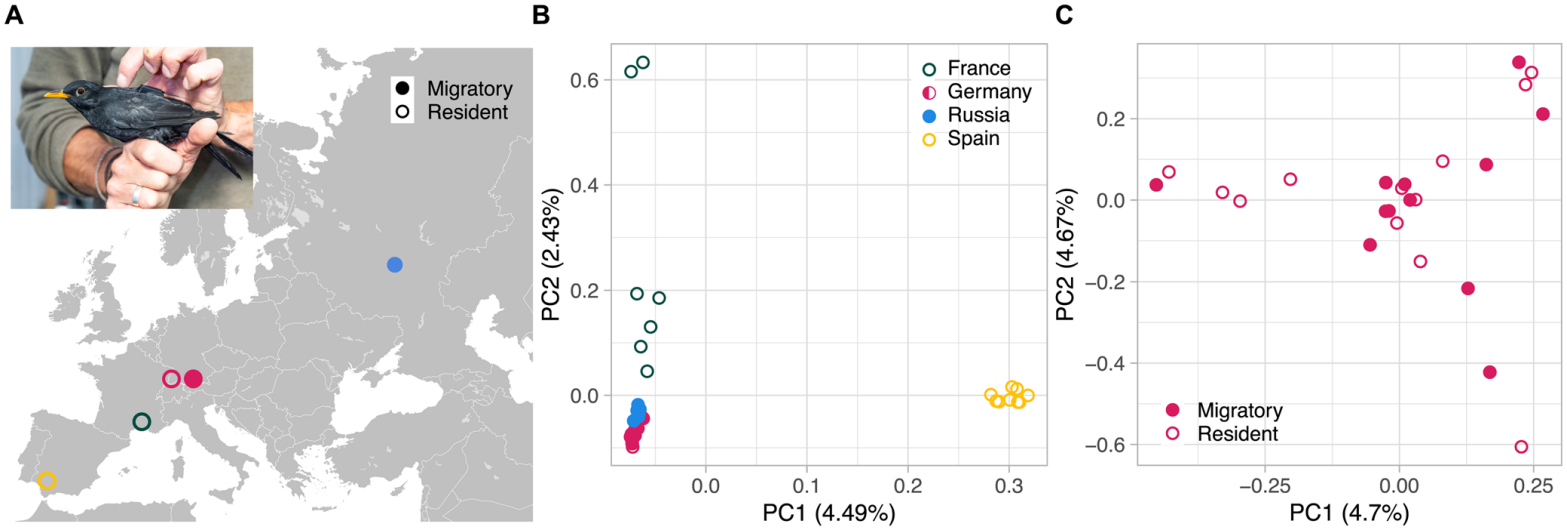
Blackbird sampling and population structure. (A) Geographic location of blackbirds sampled: Spain (resident *n* = 9), France (resident *n* = 7), Germany (resident *n* = 12, migratory *n* = 12), and Russia (migratory *n* = 9). Circles represent individuals colored according to their population, open circles indicate resident, filled circles migratory phenotypes. The photograph in the upper left corner shows a male blackbird equipped with a radio transmitter (taken by Jesko Partecke). (B) Genome‐wide principal component analysis (PCA) of SNV genotypes. Individuals from the Spanish population are clustered together, with distinct separation on the first principal component, while variation within the French population is captured mostly on the second principal component. Individuals from the German and Russian populations are tightly clustered, especially on the first principal component. (C) PCA of individuals from the German partial migratory populations with 12 resident (filled circles) and 12 migratory individuals (open circles). The lack of clear clusters illustrates that there is no population subdivision corresponding to the migratory phenotype.

Here, we focus on migratory behavior in common blackbirds ( *Turdus merula* ). Blackbirds are ideally suited for this because they show a clear pattern of migratory phenotype distribution (propensity to migrate) across their distribution range with year‐round resident populations in Southern Europe, partial migrant populations in central Europe and exclusively migratory populations in Northern and Eastern Europe (Figure 1). In addition to that, the overwintering strategy of resident and migratory individuals within the partial migratory population in Germany is highly consistent (Partecke et al. *unpublished data*). Individual migratory strategies can be accurately assessed using a tracking routine that allows to remotely follow individual blackbirds in the wild over consecutive years (Fudickar et al. 2013). To understand the genomic architecture underlying migratory strategies in this system, we here (I) assembled a high‐quality reference genome; (II) obtained behavioral phenotypes (i.e., migratory strategy) of individuals within a mixed population; (III) generated whole‐genome sequencing data of these individuals in addition to individuals of year‐round resident and fully migratory populations; and (IV) performed population genomic analyses to identify genomic candidate loci under selection. Our results show that while there is very little genetic differentiation between migratory phenotypes in the German partial migratory population–as expected in a fully panmictic population–there are a few divergent genomic regions that provide insights into the genetic architecture of the migratory phenotype, as they allow a direct association to individual behavioral phenotypes.

## 2 Materials and Methods

### 2.1 Sampling, Phenotyping, and Sequencing

We sampled adult male European blackbirds at four different study sites across the Eurasian breeding range, covering the species' migratory phenotype spectrum: 10 individuals from a breeding population in Spain (N 37° 17′, W 6° 20′) and 10 individuals from a population in France (N 44° 16′, E 4° 43′) representing year‐round residents; a partial migratory population in Germany (N 47° 46′, E 9° 2′) with 15 migratory and 15 resident birds individually phenotyped; and 10 migratory individuals breeding in Russia (N 55° 27′, E 37° 10′) (Table S1). To characterize migratory strategies of all individuals, we caught them with mist nets during spring and summer preceding autumn migration and deployed long‐lasting radio transmitters (≤ 2.6 g delivered by Sparrow Systems, Fisher, IL, USA) using the leg‐loop harness method as previously described (Fudickar et al. 2013). One to five stationary automated receivers (Sparrow Systems, Fisher, IL, USA) were deployed at each study site to continuously monitor the presence of individuals, and eventual departure dates (Crofoot et al. 2008; Kays et al. 2011). Each automated receiver searched for a maximum of 16 frequencies every 60 s. Automated receivers were connected to H antennas (ATS, Isanti, MN, USA), mounted 3–12 m high. 24‐h ARU monitoring enabled us to precisely determine individual departure events via a rapid decline of signal strength of the radio transmitters (Zúñiga et al. 2016). We used ARU data sightings and manual tracking to confirm the absence of an individual within a 2.5 km radius. Manual tracking was done via a combination of handheld H antenna (Andreas Wagener Telemetry Systems, Köln, DE) and Yaesu VR 500 receiver (Vertex Standard USA, Cypress, CA, USA). For the partial migratory German population, we also used a car‐mounted Yagi‐antenna (AF Antronics Inc., Urbana, IL, USA) and an airplane equipped with two H‐antennas and two Biotrack receivers (Lotek, Newmarket, ON, Can) to confirm departure of an individual within a 20 km radius of the study site and thus validate the 2.5 km radius, which we used to define departure events in the other study areas.

Blood samples (ca. 50–100 μL) of all individuals were collected from the brachial vein and stored in 1 mL Queen's lysis buffer or 500 μL 95% EtOH; samples were stored in a −80°C freezer until further processing. High molecular weight DNA was extracted from blood following a standard salt extraction protocol. We prepared small insert size libraries using DNA from each individual and sequenced five samples per lane on NextSeq 500 with paired‐end 150 bp reads.

### 2.2 Reference Genome

We used an adult male from the German population, originating from our in‐house breeding population and not characterized for migratory phenotype in the wild to generate a new reference genome. Blood (100 μL) was collected in 500 μL 95% EtOH, flash frozen in liquid nitrogen and stored at −80°C until further processing. High molecular weight genomic DNA was extracted using the KingFisher Cell & Tissue DNA preparation Kit (Cat# 97030196). We used a combined Pacific Biosystems (PacBio) long‐read sequencing and BioNano Genomics optical mapping strategy to generate a hybrid assembly of the blackbird reference genome. BioNano genome maps were generated using Direct Label and Stain (DLS) technology. Large insert size (15–20 kb) libraries were prepared for PacBio Sequel I Sequencing and subsequently sequenced on 20 PacBio SMRT cells. FALCON v1.9.0 and FALCON unzip v1.0.6 were used to generate haplotype phased contigs, which were then super‐scaffolded using the BioNano Irys software hybrid scaffolding pipeline (Chin et al. 2016).

We then used SatsumaSynteny (Grabherr et al. 2010) to determine to which avian chromosome each scaffold in our final assembly corresponded to. Specifically, we aligned the blackbird scaffolds to the collared flycatcher ( *Ficedula albicollis* ) assembly (Ellegren et al. 2012) and organized scaffolds by chromosome and location. We validated the final assembly searching for ultra‐conserved elements (UCEs) identified by Faircloth et al. (2012) (using whole genome alignments of the chicken and zebra finch). We downloaded the sequences for these elements and used NCBIs ‘blastn’ to determine how many were in our assembly (−evalue 1e‐20 ‐perc_identity 90).

### 2.3 Genome Annotation

We carried out a gene prediction to annotate and assign putative functions and protein domains using MAKER (Cantarel et al. 2008). Gene prediction was performed using as input a previously assembled blood transcriptome of blackbirds (18,219 assembled transcripts (Franchini et al. 2017); assembly source: https://datadryad.org/stash/dataset/doi:10.5061/dryad.tc722) and cDNA sequences from three avian species (zebra finch taeGut3.2.4, chicken Galgal4 and collared flycatcher FicAlb_1.4) obtained from the Ensembl database. After four rounds of gene prediction with MAKER, we only kept gene models with an Annotation edit Distance (AED) less than 0.5 and more than 50aa in length. The functional annotation of the predicted protein sequences was done using blastp against a database of predicted proteins from ensemble (same species as above). Protein domains and GO ontologies were annotated using Interproscan.

### 2.4 Repetitive Element Characterization and Annotation

To characterize the repetitive content of the blackbird genome, we produced a de novo repeat library of raw consensus sequences using RepeatModeler2 (with the ‐ltr_struct option) (Flynn et al. 2020). On top of RepeatModeler2, we also detected full‐length LTR retrotransposons with LTR Harvest (Ellinghaus, Kurtz, and Willhoeft 2008) and LTR Digest (Steinbiss et al. 2009) as described in (Peona, Palacios‐Gimenez, et al. 2021). The library of raw consensus sequences from RepeatModeler2 together with the avian transposable element library from Peona et al. (2021) was then used to annotate the blackbird genome with RepeatMasker v4.1.2 (Smit, Hubley, and Green 2015). The output of RepeatMasker (.align file) was further processed with the scripts calcDivergenceFromAlign.pl. from RepeatMasker to re‐calculate the divergence of insertions from their consensus sequences taking CpG sites into account for subsequent plotting of the repeat landscape.

### 2.5 Read Mapping and Variant Calling

Adaptor trimming of raw re‐sequencing fastq files was performed with *bbduk* (sourceforge.net/projects/bbmap/). We then mapped fastq files of individuals to the blackbird reference assembly *bTurMer1* using *bwa‐mem2* (Vasimuddin et al. 2019). Following sorting and indexing of alignment files, we removed duplicates with *samtools* (Danecek et al. 2011). To identify an initial set of SNVs per individual, we first used *bcftools mpileup*, specifying a minimum mapping quality of 20, and a minimum base quality of 20, followed by *bcftools call* with set to 2 (Danecek et al. 2011). The resulting gvcf files were merged per scaffold and the following filters were applied using *bcftools*: a SNV quality of above 20, a read depth of more than the total number of samples and less than twice the average read depth, mapping quality above 30, and RPBZ between −3 and 3. For sites with a read depth less than four, genotypes were set to missing. Further, we removed individuals with less than half of the genome‐wide average read depth, and SNVs with more than 20% missingness. Finally, we also removed variants that overlapped with entries of our repeat annotation using *BEDtools* (Quinlan and Hall 2010).

### 2.6 Population Genomic Analysis

The resulting SNV vcf file was used for all downstream analyses, including genome‐wide F_ST_ scans, principal component analysis (PCA), and linkage‐disequilibrium (LD) analyses. First, we converted the vcf file to bim format using *plink* (Purcell et al. 2007). We then used the R package *gdsfmt* (Zheng et al. 2012) to convert it to a geno file. Using this geno file we then performed the PCA and relatedness analysis using *SNPRelate* in R (Zheng et al. 2012). The latter enabled us to identify closely related individuals (parent‐offspring and half‐siblings) in our dataset, of which we then subsequently excluded the individual with the lower genome‐wide average read depth from each pair. To calculate genomic differentiation (F_ST_) in 2.5 kb windows, we used *popgenwindows.py* (https://github.com/simonhmartin/genomics_general), which outputs π for both populations of the comparison, as well as *D*_*xy*_ and F_ST_ (Hudson, Slatkin, and Maddison 1992). We chose this window size based on the assumption that a polygenic architecture underlies our trait of interest (migratory behavior) (Le Corre and Kremer 2012), therefore allowing a more fine‐grained investigation of allele frequency changes of SNVs aids identifying genomic regions under selection, especially in a setting in which a presumably fully admixed population contains two divergent phenotypes. To discern population genetic processes that are common in all populations (e.g., background selection) from those that are of interest in the focal population comparison (i.e., selection on the behavioral phenotype), we calculated ΔF_ST_’. Following Vijay et al. (2016), we first estimated F_ST_ for a comparison that is within the same phenotype (i.e., the two resident populations of Spain and France). We then Z‐transformed and subtracted this estimate for each orthologous 2.5 kb window from the Z‐transformed estimates of the between‐phenotype comparisons (e.g., resident vs. migratory German individuals), and presumably retain a signal indicative of divergent selection. We also calculated per‐SNV F_ST_ after Weir and Cockerham (1984) using *vcftools* (Danecek et al. 2011). To identify signatures of selection, we estimated Tajima's *D* in the same 2.5 kb windows used in the analyses above using *vcftools* (Danecek et al. 2011). To investigate the potential occurrence of an inversion on the blackbird equivalent of collared flycatcher chromosome 9 in, we calculated pairwise linkage disequilibrium (LD) among all SNVs present on that chromosome with *vcftools* (Danecek et al. 2011).

Downstream data handling and visualization have been performed with the *R* programming language, using the packages *ggplot2* and *tidyr* (Wickham, Vaughan, and Girlich 2024).

## 3 Results

### 3.1 Genome Assembly and Annotation

To produce a draft reference assembly, we generated 110.6 Gigabytes of PacBio long‐read sequencing data from 21 SMRT cells, corresponding to a 45.27‐fold genome coverage and achieving an average read length of 14 kb (10.5 kb average subread length). After assembling an initial draft reference (see Methods), we generated 4,643,798 BioNano single‐molecule maps and assembled them into consensus maps to be then used to super‐scaffold PacBio scaffolds resulting in a final reference genome of 1.01 Gb (1,012,066,560 bp) in length. This new reference assembly (*bTurMer1*, available under accession number GCA_046127255.1) consists of 125 scaffolds and with a scaffold N50 of 42.23 Mb. A total of 113 scaffolds mapped to the collared flycatcher genome (an average of four scaffolds per chromosome). The *de novo* gene annotation using both *in silico* and evidence‐based approaches resulted in the identification of 18,074 protein‐coding genes and 19,060 transcripts. Results from the UCE analysis suggest this reference is nearly complete as more than 95% of the UCEs identified in amniotes were also present (2443/2560 UCEs).

### 3.2 Repeat Library and Annotation

The *de novo* repeat library obtained with RepeatModeler2 contains a total of 430 consensus sequences (Data S1). As expected from the repeat composition of other birds (Peona, Blom, et al. 2021; Kapusta and Suh 2017; Bravo, Jonathan Schmitt, and Edwards 2021), the vast majority of consensus sequences are classified as LTR retrotransposons (197) or LINE Chicken Repeat 1 (103). The rest of the elements are classified as DNA transposons (41), SINEs (11) a few are satellite DNA monomers, while 88 sequences remain unclassified. The repeat annotation (Data S2 and S3) showed that the blackbird genome is ~11% repetitive percentage similar to many other birds (Kapusta and Suh 2017; Peona, Blom, et al. 2021) where the most common transposable elements are LINEs (3.73%) and LTR retrotransposons (3.64% of which only 8% are found to be full‐length, Data S4). While the 20% of the blackbird‐specific repeat library remains unclassified, these elements account for only 0.77% of the genome assembly. Finally, the blackbird genome shows an accumulation of LTR retrotransposons in ancient and recent times, while LINE retrotransposons show an accumulation of only older insertions (with a divergence from consensus sequence greater than 10%; Figure S1). Since the blackbird‐specific repeat library has not been manually curated, precise calculation of divergence of insertions from their consensus sequences cannot be made.

### 3.3 Population re‐Sequencing

In total, we generated whole‐genome re‐sequencing data of 60 male individuals from 4 different European populations with divergent phenotypes (Spain = 10, France = 10, Germany = 30, and Russia = 10; Figure 1A). The higher number of individuals in the German population stems from the fact that we included migrants (*n* = 15) and residents (*n* = 15) from the focal partial migratory population to facilitate direct comparison of behavioral phenotypes of the same geographical region. Mean sequencing read depth among all individuals was 15.63X, and in an initial filtering step, we excluded eight individuals that showed an average read depth of less than half of the mean across all individuals (< 7.82X, Table S1). We then mapped sequence reads to the *bTurMer1* reference genome assembly and identified single‐nucleotide variants (SNVs) in each individual. Merging and extensive filtering (see Methods) resulted in a final set of 21,408,162 variants, which were analyzed subsequently. Using these SNVs, we performed a relatedness analysis using the Method of Moments (Purcell et al. 2007) approach to identify closely related individuals. We found five pairs of individuals among the German and French samples that exhibited a kinship coefficient over 0.1 (Figure S2), which led us to exclude three individuals (one migratory individual from the German and two individuals from the French population, respectively). The total number of individuals was therefore 49 individuals (9 from Spain, 7 from France, 12 German resident individuals, 12 German migratory individuals, and 9 from Russia, Table S1).

### 3.4 Population Structure

To visualize structure across blackbird populations in Europe, we performed principal component analysis (PCA) using genotypes of all filtered SNVs as input. Figure 1B shows that genomic variation separating the Spanish individuals from the rest of the European populations is captured by the first principal component, whereas the second principal component mainly illustrates variation within the French population, with German and Russian individuals tightly clustered together. Additional principal components did not exhibit further clustering according to populations or phenotypes (Figure S3). In a PCA using only individuals from the German population, we did not observe any clustering according to the behavioral phenotypes (Figure 1C).

### 3.5 Genome‐Wide Differentiation

**FIGURE 2:**
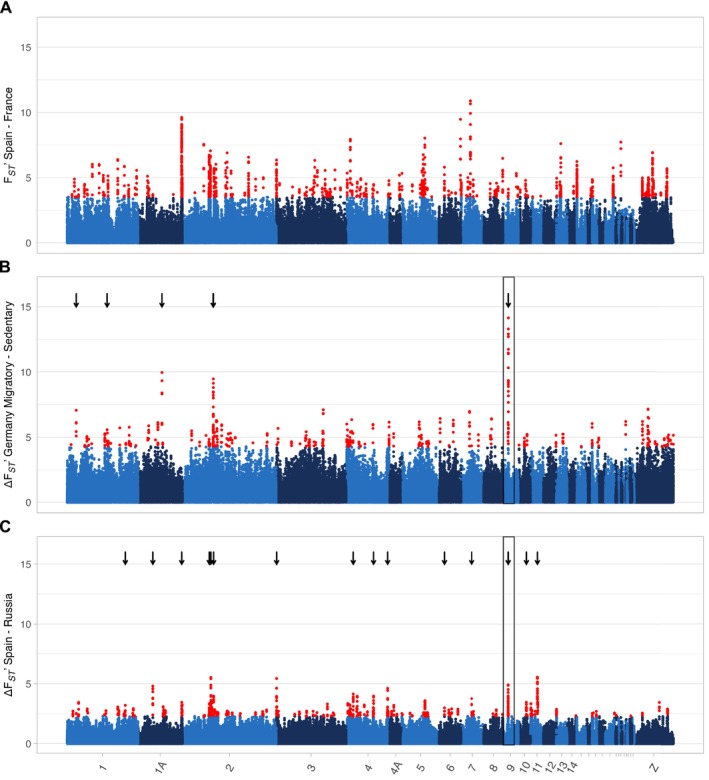
Genome‐wide differentiation scans. In (A), we compared geographically separated populations exhibiting the same migratory phenotype (Spain and France) and calculated F_ST_ in 2.5 kb windows. We then normalized these estimates by scaffold (F_ST_’) and plotted the running mean over five windows with different shades of blue corresponding to collared flycatcher chromosome models (respective chromosome number indicated on *x*‐axis in C). Windows above the 99th percentile are shown in red. (B) Comparison between migratory and resident individuals within the partial migratory population (Germany). Net differentiation (ΔF_ST_’ = F_ST_'_Spain‐France_—F_ST_'_GER migratory‐GER resident_) is plotted the same way as in (A). While the overall differentiation landscape is similarly heterogeneous compared to the within‐phenotype comparison, there is one region of markedly increased differentiation on the equivalent of collared flycatcher chromosome 9 (indicated by the rectangle). ΔF_ST_’ outlier clusters, that is, five or more consecutive outlier windows are marked by black arrows. Note that only five arrows are visible due to the close proximity of clusters on chromosomes 1, 2, and 9. (C) ΔF_ST_’ for the between‐phenotype comparison between Spain and Russia. A total of 73 outlier windows (in red) are shared between B and C, two of them also occurred in outlier cluster 1 of chromosome 9 in the between‐phenotype comparison in the German population (indicated by the rectangle).

To identify genomic regions that are associated with phenotypic differences in migration strategy, we performed F_ST_ outlier scans. For each population comparison, we calculated mean F_ST_ after Hudson, Slatkin, and Maddison (1992) in non‐overlapping windows of 2.5 kb in size and a minimum of 10 SNVs. To obtain the baseline level of differentiation presumably not related to differences in migration strategy (i.e., a ‘control’ comparison), we first focused on the within‐phenotype comparison of Spanish and French resident blackbirds. Mean genome‐wide F_ST_ in this comparison was low with 0.04 (median 0.029) ranging from 0 to 0.878 per 2.5 kb window, yet the differentiation landscape was heterogeneous across chromosomes (see Figure 2A for Z‐transformed F_ST_ estimates, Figure S4A for untransformed estimates). Next, we estimated differentiation by comparing migratory phenotypes in the German partial migratory population. To discern regions of elevated F_ST_ due to divergent selection from those due to common population processes (e.g., background selection), we Z‐transformed F_ST_ estimates (F_ST_’) and subtracted the within‐phenotype (Spain‐France) F_ST_’ from the between‐phenotype F_ST_’ to yield a measure of net differentiation (ΔF_ST_’). The genome‐wide ΔF_ST_’ landscape shows very low levels of differentiation on average, with a few regions of distinctly elevated ΔF_ST_’ (Figure 2B, Figure S4B for absolute F_ST_). Windows above the 99th percentile of ΔF_ST_’ were deemed as outliers. Overall, only seven ΔF_ST_’ outlier windows overlapped with F_ST_’ outlier windows of the ‘control’ comparison between both resident Spanish and French individuals. When five or more outlier windows occurred consecutively (ignoring windows with missing values)—indicative of selection potentially acting on haplotypes instead of single SNVs—we classified them as outlier clusters. In total, we found eight outlier clusters belonging to five regions on four chromosomes (Table 1).

**TABLE 1:** Summary of ΔF_ST_’ outlier clusters for the between‐phenotype German comparison.

Cluster ID	Windows in cluster	Mean ΔFST’	Mean FST	Median FST	Mean Tajima's D Migratory	Mean Tajima's D resident	Chromosome
Super‐Scaffold_100156_cluster401	7	11.664	0.136	0.111	−0.68	−1.688	9
Super‐Scaffold_100156_cluster405	12	10.465	0.109	0.1	−0.531	−0.882	9
Super‐Scaffold_100168_cluster14781	6	9.166	0.07	0.066	−0.254	−0.724	1A
Super‐Scaffold_100237_cluster1039	7	8.107	0.066	0.065	−0.344	−0.387	2
Super‐Scaffold_100237_cluster1044	7	8.286	0.068	0.059	−0.047	−0.14	2
Super‐Scaffold_100237_cluster944	5	5.56	0.043	0.039	−0.014	−1.279	2
Super‐Scaffold_100355_cluster12929	5	7.06	0.06	0.047	−0.268	−0.419	1
Super‐Scaffold_100355_cluster775	5	5.573	0.052	0.051	−0.389	−0.341	1

**FIGURE 3:**
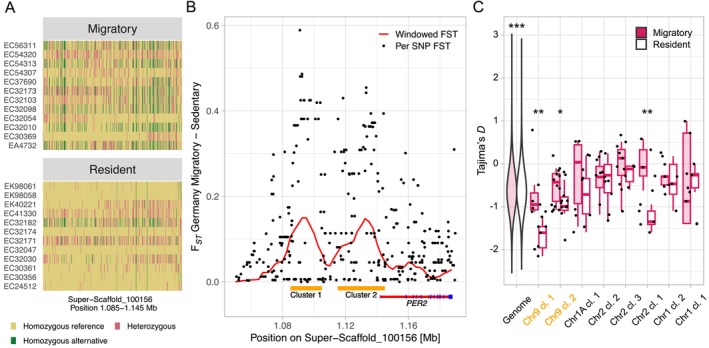
Between‐phenotype German comparison ΔF_ST_’ outlier cluster on the equivalent of flycatcher chromosome 9. (A) Haplotype plot of SNVs. Rows represent individuals, vertical tiles SNV genotypes colored according to genotype. While there are no variants fixed between the two migratory phenotypes, there is a clear separation of haplotypes visible. (B) Windowed (red line indicating the running mean) and per‐SNV F_ST_ (black dots) estimates. Yellow bars indicate the two cluster regions with 7 and 12 consecutive outlier windows, respectively. The gene model of the *PER2* gene is shown in the lower right corner. (C) Tajima's *D* estimates in 2.5 kb windows of migratory phenotypes in the German population. The two violin plots indicate the genome‐wide estimates for migratory (pink) and resident (white) phenotypes. Box‐ and jitter plots show estimates for each outlier cluster. Genome‐wide, clusters 1 and 2 on chromosome 9 (orange as in B), and cluster 3 on chromosome 2 estimates are significantly different (Kruskal–Wallis test, *p* = 1.33^−12^, 1.75^−3^, 3.77^−2^, and 9.02^−3^, respectively).

The two clusters with highest overall net and absolute differentiation, as well as the most consecutive outlier windows were located on the equivalent of collared flycatcher chromosome 9 (Table 1). This separation was also clearly visible on the haplotype structure (Figure 3A). To determine whether there are any fixed variants between the two phenotypes, we calculated F_ST_ per SNV after Weir and Cockerham (1984). Single‐SNV values in the outlier cluster region on chromosome 9 corroborated the pattern observed in the ΔF_ST_’ estimates per 2.5‐kb window (Figure 3B), however, we found no fixed (F_ST_ = 1) variant in that comparison. Notably, the largest outlier cluster region on chromosome 9 is partially overlapping the *PER2* gene (Figure 3B), and in this region we observed more heterozygous and homozygous SNVs for the alternative allele in migratory individuals (909 and 584, respectively) compared to resident ones (601 and 150, respectively).

To further investigate whether this outlier region might be under divergent selection, we estimated Tajima's *D* in 2.5 kb windows for both migratory and resident individuals in the partial migratory population in Germany. Genome‐wide, Tajima's *D* estimates were on average negative with −0.602 and −0.593 for migratory and resident individuals, respectively (Figure 3C), with this slight difference (0.985‐fold decrease in resident individuals) being significant (Kruskal–Wallis test, *p* = 1.33^−12^). When focusing on windows belonging to outlier clusters of the across‐phenotype comparison in the German population, we found significant differences in Tajima's *D* estimates in three outlier clusters (two of them belonging to the main differentiation peak on chromosome 9). Two clusters exhibited the most pronounced differences: cluster 1 on chromosome 9 with a mean Tajima's *D* value of −1.687 in resident, and −0.6801 in migratory individuals, respectively, which also exhibits the highest ΔF_ST_’ estimates, and cluster 1 on chromosome 2 (−0.013 and −1.278, respectively). While the overall negative values in both resident and migratory individuals suggest that selection might be acting on both phenotypes, these results might also indicate population expansion.

To expand beyond the within population comparison of resident and migratory individuals within the partial migratory German population, and to provide a broader perspective on the phenotypic contrast between migratory and resident individuals, we estimated ΔF_ST_’ (as described above) for the comparison between the Spanish (resident) and Russian (migratory) population (Figure 2C, Figure S4C for FST), and identified outliers and outlier clusters. We found 17 outlier clusters in total, spread over 10 chromosomes. Due to the substantial geographic distance between the two compared populations, the number of outlier clusters is expectedly increased (Table S2). While no outlier clusters were shared directly between this and the within‐Germany comparison, we found a total of 73 outlier windows shared between comparisons, of which two from the Spain–Russia comparison were also part of outlier cluster 2 on chromosome 9 in the within Germany comparison.

### 3.6 Inversion Detection

**FIGURE 4:**
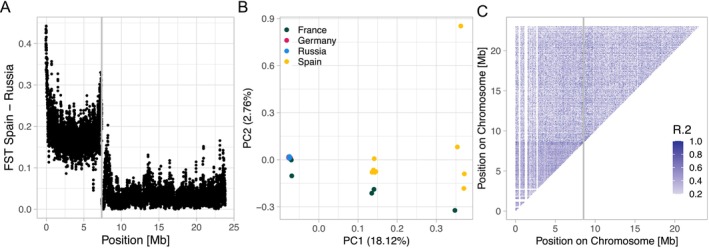
Putative inversion on chromosome 9. (A) Running mean of F_ST_ between the Spanish and Russian population in 2.5 kb windows over chromosome 9. One of the two scaffolds assigned to this chromosome shows elevated differentiation compared to the other scaffold as well as the genome‐wide average. The two scaffolds are separated by a vertical gray line. (B) PCA of individuals from all populations using only SNVs from the scaffold presumably harboring the inversion (Super‐Scaffold_100189). PC1 separates individuals into three clearly distinct clusters, with the middle and right cluster containing only individuals from the Spanish and French populations. (C) Chromosome‐wide linkage disequilibrium (LD) including individuals from all populations. The *x*‐ and *y*‐axis represent physical position on chromosome 9, and tiles are colored according to LD between SNVs. Elevated LD is only observed within one scaffold, and does not extend beyond the scaffold boundary (indicated by the gray vertical line; the inverted scaffold is shown left of the gray line).

When estimating genetic differentiation between the Spanish and other populations, we noticed a peculiar pattern on the equivalent of flycatcher chromosome 9 (see Figure 4A for the Spain–Russia comparison): One of the two scaffolds that have been assigned to collared flycatcher chromosome 9 by synteny, Super‐Scaffold_100189, exhibited markedly elevated F_ST_ estimates (4.95‐fold compared to the genome‐wide average and 5.98‐fold to the other scaffold assigned to this chromosome). This pattern is observed when comparing the Spanish and other populations, and to a lesser degree in comparisons with the French population, but is neither visible in the within‐Germany nor Germany‐Russia comparison (Figure S5A,B). While the PCA using genome‐wide SNVs showed more or less a pattern expected from geographic isolation‐by‐distance (Figure 1B), results using SNVs only from Super‐Scaffold_100189 showed a clear separation into three clusters not corresponding to geographic structure (Figure 4B). Such a distinct pattern is indicative of an inversion segregating within a population (Huang et al. 2020; Ma and Amos 2012; Todesco et al. 2020), and when including individuals from the German and Russian populations, this pattern disappears (Figure S5C), suggesting that the inversion only segregates within the Spanish and French populations. To determine whether the putative inversion might extend beyond scaffold boundaries and reach into Super‐Scaffold_100156, which harbors the main differentiation peak in the focal comparison, we estimated linkage disequilibrium (LD) over the entire chromosome (Figure 4C). Increased LD was confined to Super‐Scaffold_100189, which also showed the PCA pattern indicative of an inversion (Figure 4B) and did not extend into the other scaffold.

## 4 Discussion

Here we investigated the genetic basis of migratory behavior in a European songbird, the common blackbird, across the species' distribution range, using a newly assembled and annotated reference genome in combination with population whole‐genome re‐sequencing.

Overall population structure (Figure 1B) showed a pattern commonly seen in other pan‐European species: a divergent Iberian population forming a distinct, separated cluster and individuals from populations at higher latitudes following an isolation‐by‐distance pattern and/or clustering more tightly together. Such a pattern is usually explained by a common biogeographic history following repeated glaciation cycles (Hewitt 2000; Vijay et al. 2016). Populations at higher latitudes therefore often show lower levels of genetic diversity due to postglacial range expansions, concordant with our observations in the German and Russian populations.

When focusing on the partial migratory German population to investigate the genetic basis of migratory behavior, we found no structure or clustering according to migratory phenotypes when using genome‐wide information (Figure 1C), which suggests that the population is fully panmictic and presumably no assortative mating with respect to migratory strategy occurs. Within‐population levels of genetic differentiation (both absolute and ΔF_ST_’) between migratory phenotypes were very low, a pattern also seen in other genomic comparisons of migratory phenotypes (e.g., Delmore et al. 2020, 2023 in blackcaps). However, among the outlier clusters identified, we found one region with pronounced contrasts in both absolute and relative differentiation in 2.5 kb windows, as well as per‐SNV F_ST_. This region, located on the equivalent of the collared flycatcher chromosome 9, overlaps with the *PER2* gene (Figure 3B), a member of the *clock* gene family and known to be influential in the regulation of the circadian clock (Cassone 2014). Besides influencing regulation and entrainment of the circadian rhythm, *clock* genes have also been shown to affect the circannual rhythm, orchestrating not only reproductive cycles, but also behavioral and physiological changes related to migratory behavior. In a previous study of the same partial migratory population, Franchini et al. (2017) have found *PER2* to be differentially expressed in blood samples between two groups of migratory individuals that differed in the timespan between sampling and onset of migration (8–9 days vs. 14–18 days). Furthermore, Louder et al. (2024) have found another gene of the *clock* family, *PER3*, to be differentially expressed between migratory states in Swainson's thrushes in all brain regions. These results corroborate the important role of the *clock* genes in orchestrating and controlling migratory behavior, making a causal relationship between the differentiated region identified in our study and the propensity to migrate plausible. While we did not find any variants fully associated with the phenotype, that is, fixed SNVs, this genomic locus might still be relevant for the regulation of migratory behavior, as it can be expected that a complex behavioral phenotype is controlled by many or at least multiple variants (as shown in e.g., honey bees, Page, Rueppell, and Amdam 2012). While speculative, the fact that we found more homozygous SNVs for the alternative allele in that region potentially indicates an influence of allele number on the expression of *PER2*. Other outlier clusters were also located in or nearby genes of potential relevance for the migratory phenotype (e.g., the outlier cluster on chromosome 2 overlaps with the *TPK1* gene, which is involved in regulating metabolism). While these regions were highlighted as ΔF_ST_’ outlier clusters, their absolute differentiation is substantially lower compared to the outliers on chromosome 9 (see Table 1), suggesting that either selection acting on these regions might not have been as strong, or ΔF_ST_’ might have been artificially increased due to the very low overall differentiation.

Genome‐wide estimates of Tajima's *D* were negative in both phenotypes of the German population, which is commonly interpreted as an indication of recent population expansion (Peart et al. 2020), and is concordant with findings in other songbird populations in higher latitudes (Poelstra et al. 2014). Although these genome‐wide estimates were significantly lower in resident individuals, the difference was minute and likely not biologically relevant. In some of the ΔF_ST_’ outlier clusters however, particularly on chromosome 9, the decrease in Tajima's *D* in resident individuals was much more pronounced, which is possibly an indication of purifying selection (Tajima 1989; Jackson, Campos, and Zeng 2015).

On chromosome 9, we detected a putative inversion present on one of the two scaffolds assigned to this chromosome (different to the one harboring the F_ST_ outlier region). Our F_ST_ analysis suggests that the inversion segregates only in the Spanish and French populations (Figure 4A), which is supported by the PCA pattern when using individuals of all populations (Figure 4B). While the inversion does not seem to extend to the outlier region also present on the same chromosome (Figure 4C), its presence is peculiar. It is possible that there is a cline in the inversion frequency, however more sampling is necessary to support that. Segregating inversions are common in birds, with sometimes demonstrated phenotypic consequences (Sanchez‐Donoso et al. 2022; Hooper and Price 2017; Knief et al. 2016), yet other times seemingly without any phenotypic or fitness effects at all. Since the assignment to the chromosome is in this case solely by synteny to other bird species, it is also possible that the scaffold harboring the inversion indeed belongs to another chromosome.

In conclusion, our results present a first step toward understanding the genetic architecture that leads to the partial migratory phenotype in a songbird species. While the region with elevated genetic differentiation on chromosome 9 overlapping the *PER2* gene certainly does not provide proof for a genetic control of the migration behavior, it can be assumed that genetic variants present in that region contribute to the manifestation of migratory phenotypes, especially considering a polygenic control. The next step is now to take this knowledge to build an experimental framework that involves breeding and crossing of known phenotypes to tease apart the genetic underpinnings of this behavior.

## Author Contributions

**Matthias H. Weissensteiner:** data curation (lead), formal analysis (lead), investigation (equal), validation (lead), visualization (lead), writing – original draft (equal). **Kira Delmore:** conceptualization (equal), formal analysis (supporting), investigation (supporting), writing – review and editing (supporting). **Valentina Peona:** formal analysis (supporting), visualization (supporting), writing – review and editing (supporting). **Juan Sebastian Lugo Ramos:** formal analysis (supporting), writing – review and editing (supporting). **Gregoire Arnaud:** methodology (supporting), writing – review and editing (supporting). **Julio Blas:** methodology (supporting), writing – review and editing (supporting). **Bruno Faivre:** methodology (supporting), writing – review and editing (supporting). **Ivan Pokrovsky:** methodology (supporting), writing – review and editing (supporting). **Martin Wikelski:** conceptualization (equal), investigation (equal), methodology (equal), project administration (equal). **Jesko Partecke:** conceptualization (equal), investigation (equal), methodology (lead), project administration (equal), writing – review and editing (supporting). **Miriam Liedvogel:** conceptualization (lead), funding acquisition (lead), investigation (lead), project administration (lead), supervision (equal), writing – original draft (equal).

## Conflicts of Interest

The authors declare no conflicts of interest.

## Supporting information


Data S1.

## Data Availability

Whole‐genome sequencing data produced during this project is available at the European Nucleotide Archive under project ID PRJEB76551 (accessions for each individual in Table S1).
