# Supplementary material for: Combining Individual‐Based Radio‐Tracking With Whole‐Genome Sequencing Data Reveals Candidate for Genetic Basis of Partial Migration in a Songbird
Source: Ecol Evol. 2025 Jan 9;15(1):e70800. doi: 10.1002/ece3.70800 (PMC11717897; doi:10.1002/ece3.70800)
Supplement: Supplementary file 1 — Data S1. [file ECE3-15-e70800-s001.zip › ECE-2024-08-01762_Weissensteiner_et_al_Supplementary Material.pdf]

Supplementary Figures

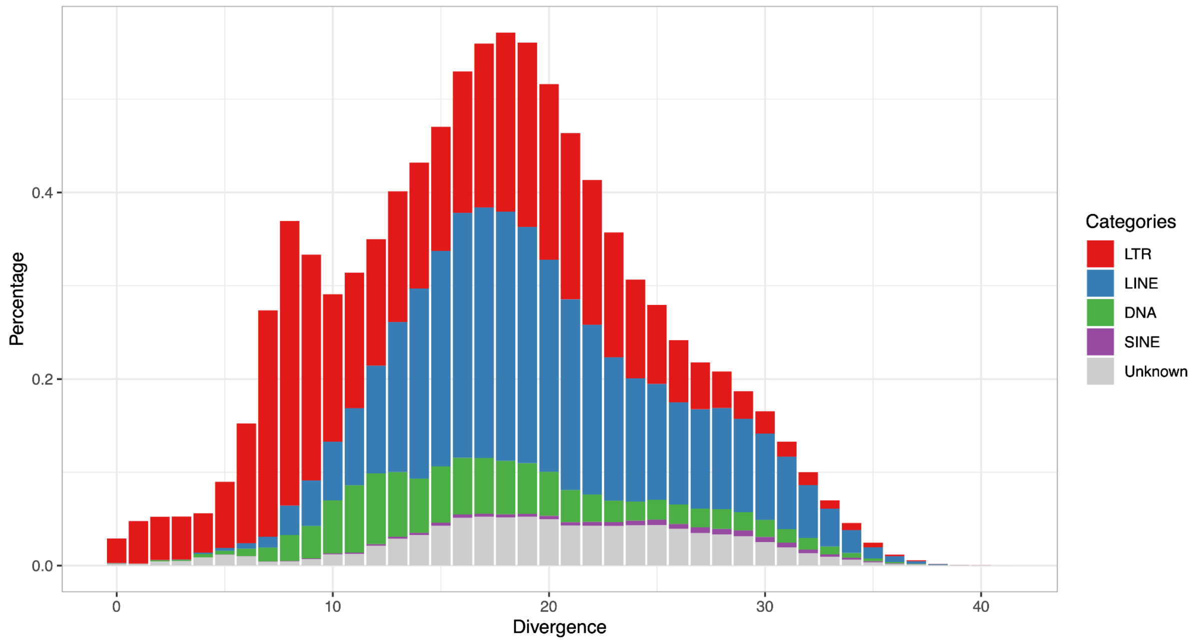

Supplementary Figure S1 Transposable element landscape of the blackbird genome as stacked bar plots. Abundance of interspersed repeats (percentage) plotted against percentage of divergence calculated as Kimura 2-parameter distance to consensus.

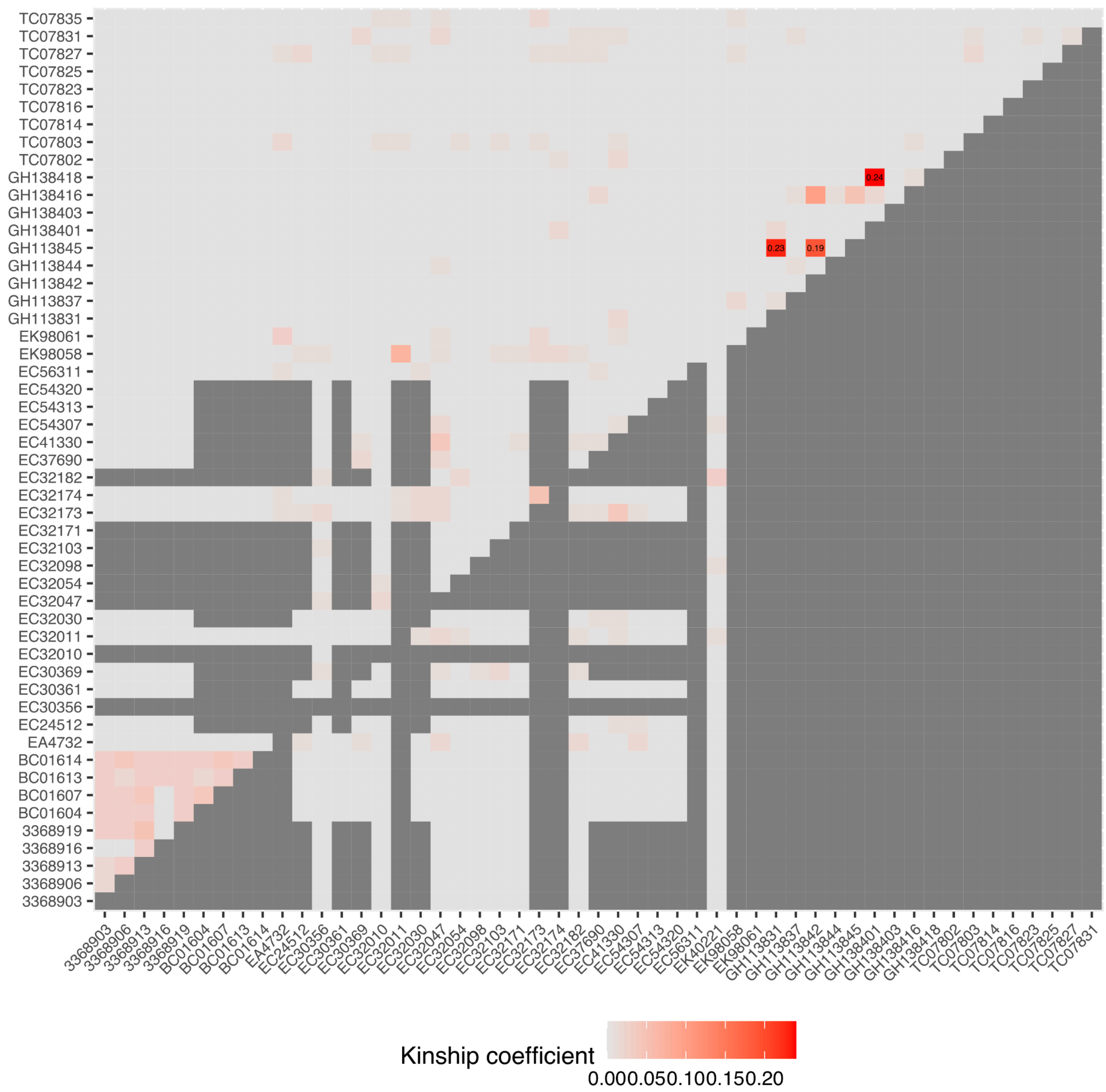

Supplementary Figure S2 Relatedness matrix of all individuals. Fill color of tiles corresponds to the Kinship coefficient calculated using the Method of Moments (Purcell et al. 2007). We found 4 pairs of individuals that exceeded a Kinship of 0.1, corresponding to at least half-siblings (1 in the German and 3 in the French population, respectively). Of these pairs, we then subsequently removed one individual that showed the lower sequencing read depth. Among the individuals closely related in the French population, one individual showed increased Kinship coefficient to two others, therefore only two individuals needed to be removed.

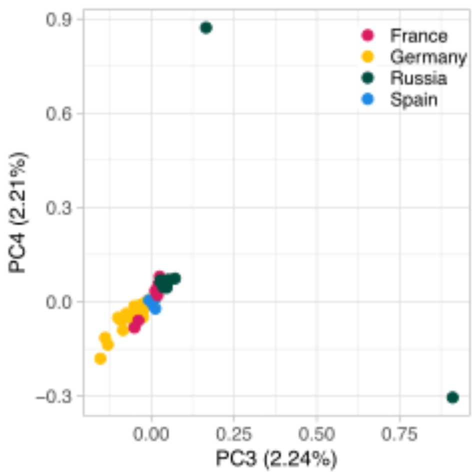

Supplementary Figure S3 Genome-wide principal component analysis (PCA) of SNV genotypes, using principal components 3 and 4 for the x- and y-axis, respectively. Individuals from all populations are all more or less tightly clustered together, exhibiting no structure according to geographic origin.

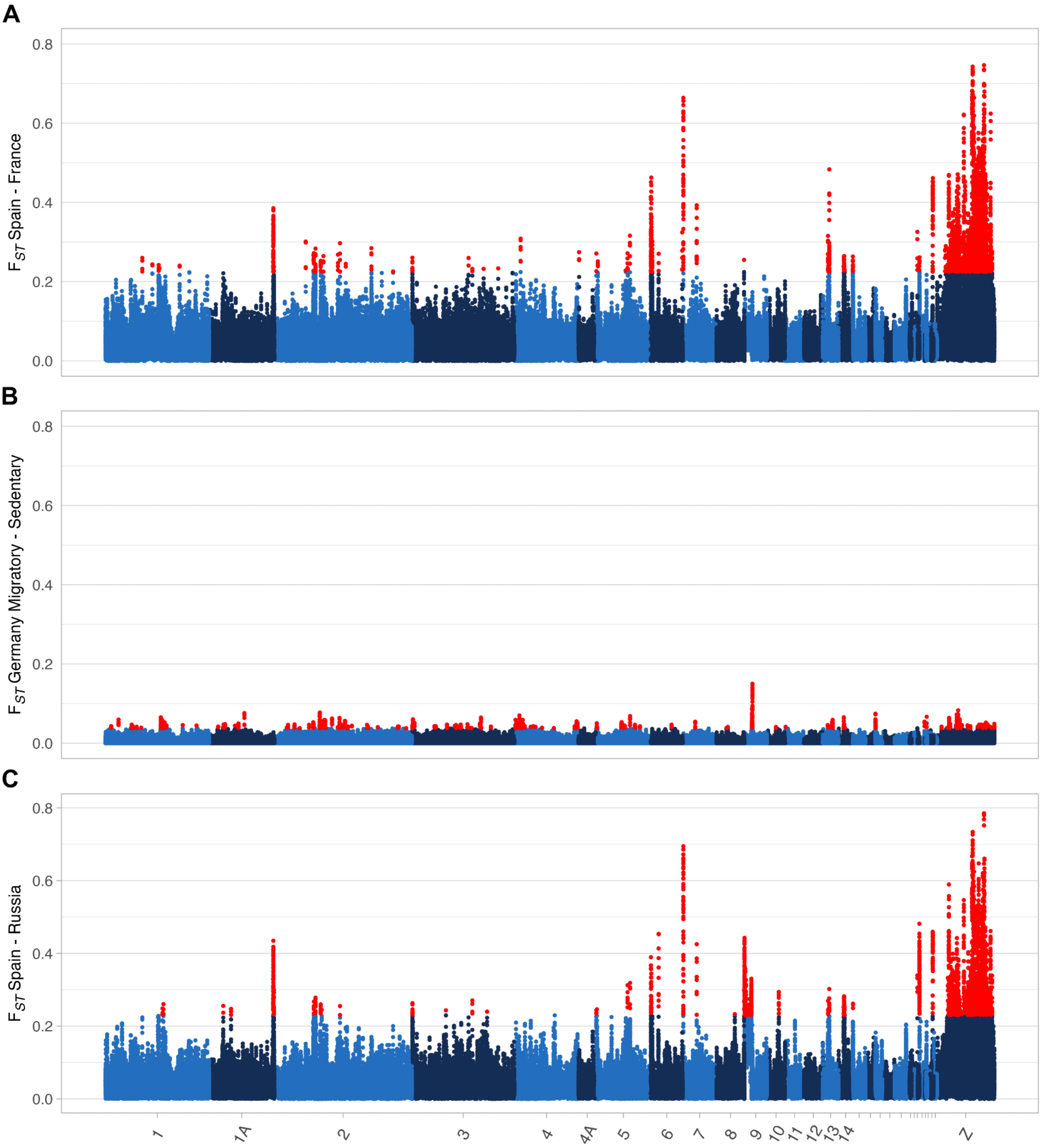

Supplementary Figure S4 Genome-wide differentiation scans. In A), we compared geographically separated populations exhibiting the same migratory phenotype (Spain and France) and calculated F_{ST} in 2.5 kb windows and plotted the running mean over five windows with different shades of blue corresponding to collared flycatcher chromosome models (respective chromosome number indicated on X axis in C). Windows above the 99th percentile are shown in red. B) Comparison between migratory and resident individuals within the partial migratory population (Germany). C) F_{ST} for the between-phenotype comparison between Spain and Russia.

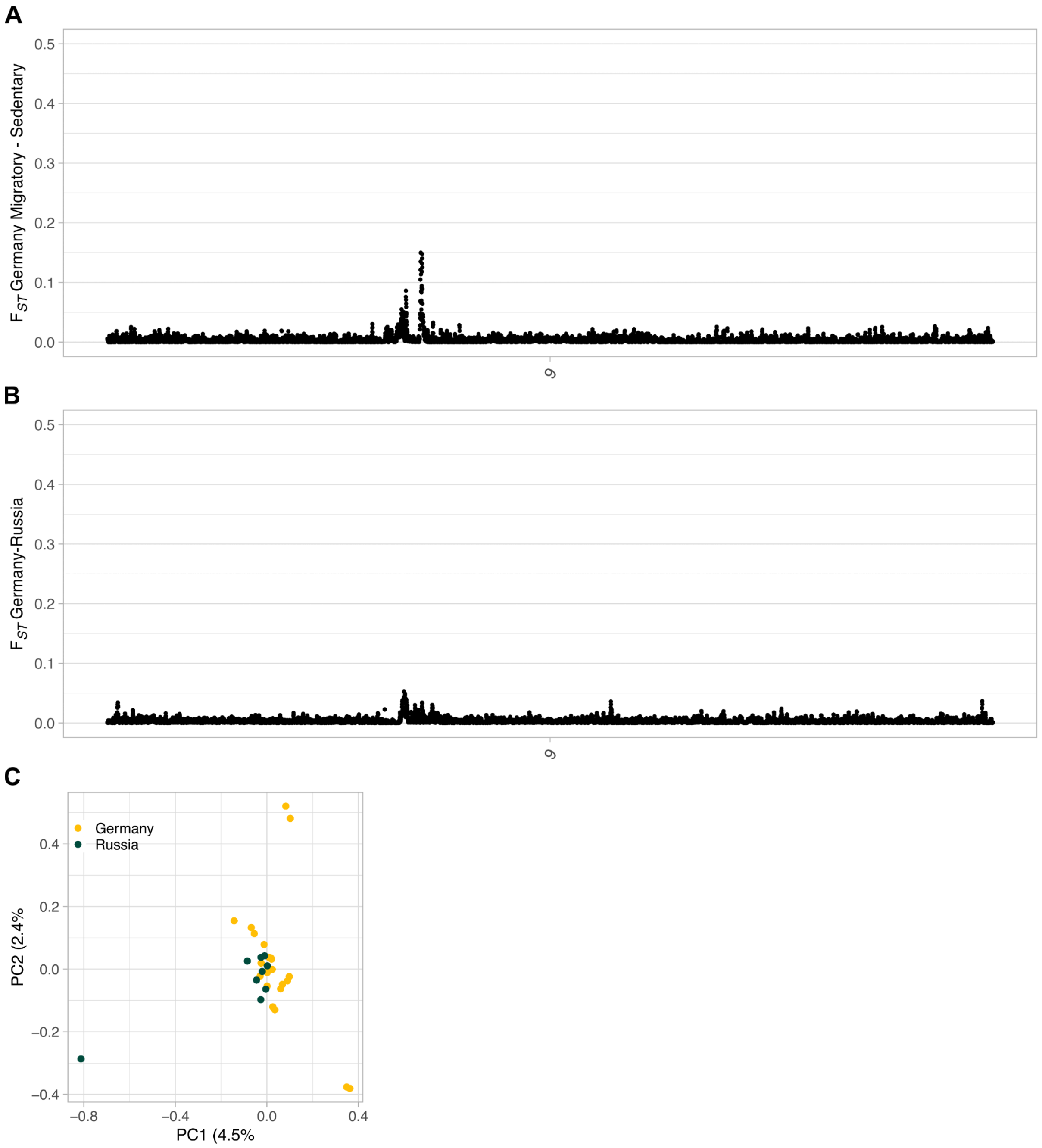

Supplementary Figure S5 Putative inversion on collared flycatcher chromosome 9. Shown in A) and B) is the running mean of absolute F_{ST} between migratory phenotypes within Germany and Germany and Russia, respectively. In contrast to the pattern observed in the Spain-Russia comparison (a pronounced increase of F_{ST} in the first scaffold, **Figure 5A**), the overall level of F_{ST} stays the same over the entire chromosome, except for the differentiation peaks on the second scaffold (discussed in **Figure 4**).

Supplementary Table S1. Samples and Sequencing depth. Bird IDs in bold indicate individuals that have been used in the downstream analys

Bird ID	Population	Coordinates	Migratory Phenotype	Mean Sequencing Read Depth	BioSample	Run accession
**3368903**	Spain	N 37° 17', W 6° 20'	Resident	19.1	SAMEA115729032	ERR13261399
**3368906**	Spain	N 37° 17', W 6° 20'	Resident	19.7	SAMEA115729033	ERR13261400
**3368913**	Spain	N 37° 17', W 6° 20'	Resident	15.8	SAMEA115729034	ERR13261401
**3368916**	Spain	N 37° 17', W 6° 20'	Resident	15.4	SAMEA115729035	ERR13261402
**3368919**	Spain	N 37° 17', W 6° 20'	Resident	17.2	SAMEA115729036	ERR13261403
**BC01604**	Spain	N 37° 17', W 6° 20'	Resident	14.1	SAMEA115729037	ERR13261404
**BC01607**	Spain	N 37° 17', W 6° 20'	Resident	13.9	SAMEA115729038	ERR13261405
**BC01613**	Spain	N 37° 17', W 6° 20'	Resident	20.2	SAMEA115729039	ERR13261406
**BC01614**	Spain	N 37° 17', W 6° 20'	Resident	16.7	SAMEA115729040	ERR13261407
BC01615	Spain	N 37° 17', W 6° 20'	Resident	5.0	SAMEA115729041	ERR13261408
**EA4732**	Germany	N 47° 46’, E 9° 2’	Migratory	20.1	SAMEA115729042	ERR13261409
**EC24512**	Germany	N 47° 46’, E 9° 2’	Resident	20.3	SAMEA115729043	ERR13261410
**EC30356**	Germany	N 47° 46’, E 9° 2’	Resident	16.6	SAMEA115729044	ERR13261411
**EC30361**	Germany	N 47° 46’, E 9° 2’	Resident	9.7	SAMEA115729045	ERR13261412
**EC30369**	Germany	N 47° 46’, E 9° 2’	Migratory	22.8	SAMEA115729046	ERR13261413
**EC32010**	Germany	N 47° 46’, E 9° 2’	Migratory	17.3	SAMEA115729047	ERR13261414
EC32011	Germany	N 47° 46’, E 9° 2’	Resident	18.9	SAMEA115729048	ERR13261415
EC32015	Germany	N 47° 46’, E 9° 2’	Resident	18.3	SAMEA115729049	ERR13261416
EC32019	Germany	N 47° 46’, E 9° 2’	Migratory	5.4	SAMEA115729050	ERR13261417
**EC32030**	Germany	N 47° 46’, E 9° 2’	Resident	25.8	SAMEA115729051	ERR13261418
**EC32047**	Germany	N 47° 46’, E 9° 2’	Resident	19.5	SAMEA115729052	ERR13261419
**EC32054**	Germany	N 47° 46’, E 9° 2’	Migratory	17.2	SAMEA115729053	ERR13261420
**EC32098**	Germany	N 47° 46’, E 9° 2’	Migratory	14.8	SAMEA115729054	ERR13261421
**EC32103**	Germany	N 47° 46’, E 9° 2’	Migratory	14.2	SAMEA115729055	ERR13261422
**EC32171**	Germany	N 47° 46’, E 9° 2’	Resident	16.7	SAMEA115729056	ERR13261423

**EC32173**	Germany	N 47° 46’, E 9° 2’	Migratory	22.1	SAMEA115729057	ERR13261424
**EC32174**	Germany	N 47° 46’, E 9° 2’	Resident	20.1	SAMEA115729058	ERR13261425
**EC32182**	Germany	N 47° 46’, E 9° 2’	Resident	16.4	SAMEA115729059	ERR13261426
**EC37690**	Germany	N 47° 46’, E 9° 2’	Migratory	19.1	SAMEA115729060	ERR13261427
EC41329	Germany	N 47° 46’, E 9° 2’	Resident	5.5	SAMEA115729061	ERR13261428
**EC41330**	Germany	N 47° 46’, E 9° 2’	Resident	22.2	SAMEA115729062	ERR13261429
**EC54307**	Germany	N 47° 46’, E 9° 2’	Migratory	13.3	SAMEA115729063	ERR13261430
EC54312	Germany	N 47° 46’, E 9° 2’	Resident	8.5	SAMEA115729064	ERR13261431
**EC54313**	Germany	N 47° 46’, E 9° 2’	Migratory	11.7	SAMEA115729065	ERR13261432
**EC54320**	Germany	N 47° 46’, E 9° 2’	Migratory	10.9	SAMEA115729066	ERR13261433
**EC56311**	Germany	N 47° 46’, E 9° 2’	Migratory	16.9	SAMEA115729067	ERR13261434
EC56316	Germany	N 47° 46’, E 9° 2’	Migratory	7.2	SAMEA115729068	ERR13261435
**EK40221**	Germany	N 47° 46’, E 9° 2’	Resident	14.7	SAMEA115729069	ERR13261436
**EK98058**	Germany	N 47° 46’, E 9° 2’	Resident	22.1	SAMEA115729070	ERR13261437
**EK98061**	Germany	N 47° 46’, E 9° 2’	Resident	15.7	SAMEA115729071	ERR13261438
**GH113831**	France	N 44° 16', E 4° 43'	Resident	15.5	SAMEA115729072	ERR13261439
**GH113837**	France	N 44° 16', E 4° 43'	Resident	19.4	SAMEA115729073	ERR13261440
**GH113842**	France	N 44° 16', E 4° 43'	Resident	17.7	SAMEA115729074	ERR13261441
**GH113844**	France	N 44° 16', E 4° 43'	Resident	13.4	SAMEA115729075	ERR13261442
GH113845	France	N 44° 16', E 4° 43'	Resident	15.2	SAMEA115729076	ERR13261443
**GH138401**	France	N 44° 16', E 4° 43'	Resident	19.9	SAMEA115729077	ERR13261444
**GH138403**	France	N 44° 16', E 4° 43'	Resident	10.0	SAMEA115729078	ERR13261445
GH138414	France	N 44° 16', E 4° 43'	Resident	5.0	SAMEA115729079	ERR13261446
**GH138416**	France	N 44° 16', E 4° 43'	Resident	17.5	SAMEA115729080	ERR13261447
GH138418	France	N 44° 16', E 4° 43'	Resident	12.2	SAMEA115729081	ERR13261448
**TC07802**	Russia	N 55° 27', E 37° 10'	Migratory	19.3	SAMEA115729086	ERR13261453
**TC07803**	Russia	N 55° 27', E 37° 10'	Migratory	23.1	SAMEA115729087	ERR13261454
TC07813	Russia	N 55° 27', E 37° 10'	Migratory	3.4	SAMEA115729088	ERR13261455

**TC07814**	Russia	N 55° 27', E 37° 10'	Migratory	15.4	SAMEA115729089	ERR13261456
**TC07816**	Russia	N 55° 27', E 37° 10'	Migratory	14.7	SAMEA115729090	ERR13261457
**TC07823**	Russia	N 55° 27', E 37° 10'	Migratory	16.0	SAMEA115729091	ERR13261458
**TC07825**	Russia	N 55° 27', E 37° 10'	Migratory	14.1	SAMEA115729092	ERR13261459
**TC07827**	Russia	N 55° 27', E 37° 10'	Migratory	19.0	SAMEA115729093	ERR13261460
**TC07831**	Russia	N 55° 27', E 37° 10'	Migratory	19.5	SAMEA115729094	ERR13261461
**TC07835**	Russia	N 55° 27', E 37° 10'	Migratory	19.2	SAMEA115729095	ERR13261462

Supplementary Table S2. Summary of ΔFST’ outlier clusters for the comparison between Spain and Russia.							
**Cluster ID**	**Windows in cluster**	**Mean ΔFST’**	**Mean FST**	**Median FST**	**Mean Tajima's D Migratory**	**Mean Tajima's D Sedentary**	**Chromosome**
Super-Scaffold_100001_cluster42395	5	5.441	0.238	0.231	-0.17	-1.293	3
Super-Scaffold_100002_cluster1097	5	3.282	0.114	0.112	0.192	-1.402	2
Super-Scaffold_100002_cluster165	12	4.416	0.161	0.172	0.673	-0.601	2
Super-Scaffold_100003_cluster219	5	3.973	0.135	0.129	-0.825	-0.419	4
Super-Scaffold_100003_cluster6498	5	4.622	0.143	0.14	0.345	-0.182	4
Super-Scaffold_100107_cluster12045	9	3.583	0.182	0.176	0.622	-0.944	4
Super-Scaffold_100156_cluster298	6	3.988	0.13	0.116	0.146	-1.063	9
Super-Scaffold_100156_cluster307	7	4.66	0.164	0.156	-1.367	-1.311	9
Super-Scaffold_100156_cluster310	7	3.611	0.179	0.179	-0.945	-1.103	9
Super-Scaffold_100166_cluster1253	5	3.003	0.264	0.255	-1.218	-0.72	10
Super-Scaffold_100168_cluster27099	5	3.46	0.435	0.473	0.61	-0.526	1A
Super-Scaffold_100168_cluster9106	7	4.548	0.24	0.253	0.407	-0.965	1A
Super-Scaffold_100172_cluster2294	18	4.552	0.188	0.176	0.217	-0.434	11
Super-Scaffol	5	3.764	0.198	0.184	-0.632	-0.677	7

d_100173_cluster5894							
Super-Scaffold_100237_cluster1227	5	3.977	0.186	0.176	0.634	-0.879	2
Super-Scaffold_100279_cluster8557	5	3.201	0.156	0.162	-0.095	-0.402	1
Super-Scaffold_100300_cluster1047	5	2.988	0.453	0.468	-0.827	-1.443	6
